# Copy number variation in plasma as a tool for lung cancer prediction using Extreme Gradient Boosting (XGBoost) classifier

**DOI:** 10.1111/1759-7714.13204

**Published:** 2019-11-06

**Authors:** Daping Yu, Zhidong Liu, Chongyu Su, Yi Han, XinChun Duan, Rui Zhang, Xiaoshuang Liu, Yang Yang, Shaofa Xu

**Affiliations:** ^1^ Thoracic Surgery Department, Beijing Chest Hospital Capital Medical University; Beijing Tuberculosis and Thoracic Tumor Research Institute Beijing China; ^2^ Ping An Health Technology Beijing China; ^3^ Beijing Gencode Diagnostics Laboratory Beijing China

**Keywords:** cfDNA, CNV, early diagnosis, lung cancer, XGBoost

## Abstract

**Background:**

The main cause of cancer death is lung cancer (LC) which usually presents at an advanced stage, but its early detection would increase the benefits of treatment. Blood is particularly favored in clinical research given the possibility of using it for relatively noninvasive analyses. Copy number variation (CNV) is a common genetic change in tumor genomes, and many studies have indicated that CNV‐derived cell‐free DNA (cfDNA) from plasma could be feasible as a biomarker for cancer diagnosis.

**Methods:**

In this study, we determined the possibility of using chromosomal arm‐level CNV from cfDNA as a biomarker for lung cancer diagnosis in a small cohort of 40 patients and 41 healthy controls. Arm‐level CNV distributions were analyzed based on z score, and the machine‐learning algorithm Extreme Gradient Boosting (XGBoost) was applied for cancer prediction.

**Results:**

The results showed that amplifications tended to emerge on chromosomes 3q, 8q, 12p, and 7q. Deletions were frequently detected on chromosomes 22q, 3p, 5q, 16q, 10q, and 15q. Upon applying a trained XGBoost classifier, specificity and sensitivity of 100% were finally achieved in the test group (12 patients and 13 healthy controls). In addition, five‐fold cross‐validation proved the stability of the model. Finally, our results suggested that the integration of four arm‐level CNVs and the concentration of cfDNA into the trained XGBoost classifier provides a potential method for detecting lung cancer.

**Conclusion:**

Our results suggested that the integration of four arm‐level CNVs and the concentration from of cfDNA integrated withinto the trained XGBoost classifier could become provides a potentially method for detecting lung cancer detection.

**Key points:**

**Significant findings of the study**:

- Healthy individuals have different arm‐level CNV profiles from cancer patients.
- Amplifications tend to emerge on chromosome 3q, 8q, 12p, 7q and deletions tend to emerge on chromosome 22q, 3p, 5q, 16q, 10q, 15q.

**What this study adds**:

- CfDNA concentration, arm 10q, 3q, 8q, 3p, and 22q are key features for prediction.
- Trained XGBoost classifier is a potential method for lung cancer detection.

## Introduction

According to Cancer Statistics in China (2015), lung cancer (LC) is the most and second most common cancer in men and women, respectively. It is also the leading cause of cancer death in both sexes.^1^ It is believed that lung cancer is attributable to a wide range of risk factors including smoking, air pollution, environmental exposure and DNA mutation.^2^ Small cell lung cancer (SCLC) and non‐small cell lung cancer (NSCLC) are the two main types of lung cancer, with NSCLC accounting for approximately 85% of total lung cancer cases.^3^ Although TNM staging is crucial for determining feasible therapy, NSCLC patients with only early stages (I to II) could better benefit more from a comprehensive treatment based on surgery.^4^ Depending on the stage and affected region, five‐year survival rates for lung cancer range from 4% to 17%.^5^ It has been reported that survival rates decreased from 55.1% at stage I to 4.2% in cases diagnosed at stage IV;^6^ thus early detection could effectively prevent or delay disease progression.

Technological advances in the analysis of circulating tumor cells (CTCs), circulating tumor DNA (ctDNA), and tumor‐derived exosomes, which are cancer signatures in the blood, have promoted liquid biopsy as a routine diagnostic method.^7^ As one form of noninvasive liquid biopsy, ctDNA tests only require a few milliliters of blood from patients, making it easier and less expensive to obtain test samples.^8^ These 140–170 bp DNA fragments can reflect specific sequence alterations in circulating plasma.^9^ Chromosomal structural instability and copy number variations (CNVs) have been observed in almost all kinds of cancers and these recurrent alterations are associated with particular cancer types.^10^, ^11^ Cristiano *et al*.^12^ promoted an approach based on evaluating fragmentation patterns of cfDNA across the genome, finding healthy individuals had different fragmentation profiles from cancer patients. They suggested it could be broadly applied for the screening and management of patients with cancer. Ni *et al*.^13^ analyzed reproducible CNV patterns among single circulating tumor cells of lung cancer patients, and found that all eight CTCs of one patient exhibited reproducible gain and loss CNV patterns. The reproducibility of CNVs from cell to cell suggests that they are the key events of tumor metastasis. An increasing number of studies have focused on CNVs presenting in cell‐free DNA (cfDNA) fragments, which are potential biomarkers, not only for early cancer detection but also for the response to treatment and disease recurrence.^14^ Xia *et al*.^15^ evaluated CNVs in cfDNA from the plasma of lung adenocarcinoma patients and normal controls, using plasma genomic abnormality (PGA) score. They found that the PGA score of patients (19.50) was significantly higher than that of normal controls (9.28), suggesting that the alterations observed in plasma could distinguish early stage cancer in combination with other existing screening strategies. Du *et al*.^16^ analyzed CNVs of SCLC from cfDNA, and found widespread somatic CNVs among tumor related genes such as *TP53, MYC, FGFR1*, and *SOX2*. Their results demonstrated the potential clinical utility of cfDNA based liquid biopsy to SCLC early detection.

In this study, we focused on lung cancer, the main cause of cancer mortality in China and globally, and compared CNVs in cfDNA from NSCLC patients and those from normal controls, with the aim of evaluating the possibility of using cfDNA CNVs for early cancer detection.

## Methods

### Patient recruitment

Lung cancer patients who had undergone a computed tomography (CT) scan, together with histological and immunohistochemical tests at Beijing Chest Hospital between January and February in 2018 were recruited after the provision of informed consent. Briefly, patients were eligible if they were between 20 and 80 years old, with histologically‐ and immunohistochemically‐confirmed lung cancer. Patients with other cancer(s) were excluded from this study. The criteria for the normal control groups included: (i) self‐reported absence of existing or previous cancer symptoms and (ii) negative results confirmed on CT scan.

### DNA extraction, library preparation and sequencing

Peripheral blood was stored in EDTA‐containing tubes and centrifuged at 1600 × g and subsequently at 16 000 × g for 10 minutes at 4°C within six hours in order to remove the cells. DNA was extracted using 1 mL of plasma from each sample and quantified via Qubit 2.0 (Life Technology). Whole genome libraries were constructed following the instructions of the DNA NGS Library Preparation Kit (ScreenDx). After adaptor ligation, DNA enrichment and index addition were performed under 12 cycle PCR using Q5 High‐Fidelity 2× Master Mix (NEB). Agilent 2100 Bioanalyzer (Agilent) and Kapa Library Quantification Kit (Kapa Biosystems) were used to analyze and quantify the purified libraries, aiming to ensure uniform pooling before sequencing on a HiSeq X Ten sequencer (Illumina) at PE150.

### Mapping of sequencing data

Base calling and data filtration were first performed before separating raw data from each sample according to the eight‐base sequencing index. The nonrepeat‐masked human reference genome (NCBI build37/hg19) was used as reference genome to align the first single‐end reads. BWA was used as mapping tool in this step under the condition of allowing two mismatches to identify reads that mapped to a unique genomic location.

### Copy number variation detection

For the detection of CNVs, the reference genome was first divided into 100 kb windows and the number of reads falling into each 100 kb window counted. GC correction was then performed ahead of calculating chromosome arm‐level reads counts. Specifically, we summed the values of all of the 100 kb windows to obtain the read count for each chromosome arm. Finally, we applied *z* score to analyze the CNVs of each chromosome arm in accordance with the previous study.^17^ In comparison with normal controls, a *z* score higher than 2.96 represented a significant increase while a *z* score < lower than −2.96 represented a significant decrease.

### Extreme Gradient Boosting machine classifier

Extreme Gradient Boosting (XGBoost) is used for supervised learning problems, and here we used it to classify LC patients and normal individuals. XGBoost has excellent scalability and a high running speed, which have made it a successful machine learning method.^18^ In this study, tree booster was used for each iteration. To control the complexity of the model and help avoid overfitting, the L2 regularization term was applied and the maximum depth was set to three. Six vital variables were then selected for further modeling. A total of 56 samples (28 normal controls and 28 LC patients) were randomly selected to set up a training group, and the remaining 25 samples (12 normal controls and 13 LC patients) were selected as a test group. Two groups were divided randomly by the function of train_test_split in python. The area under the curve (AUC) was generated using the Scikit‐learn (sklearn) in python. Five‐fold cross validation based on the whole data set was used for further test the model stabilization. In addition, to explain the output of our machine‐learning model, we used SHapley Additive exPlanations (SHAP) values, to help us understand how a single feature affects the output of the model.^19^

## Results

### Clinical information and sequencing data

A total of 81 individuals were recruited in this study, including lung cancer patients (*N* = 41) at stage I (*N* = 20), stage II (*N* = 6), stage III (*N* = 11) and stage IV (*N* = 4), as well as normal controls (*N* = 40). Summaries of cohort and sequencing are listed in Table 1, and detailed information is listed in Table S1. The age of the cancer patients ranged from 44 to 75 (mean ~60), while it ranged from 21 to 66 (mean ~31) among normal controls. CfDNA was extracted from the plasma of all patients and normal controls. As expected, the highest cfDNA concentration appeared in cancer patients at stage IV (0.14 ng of cfDNA in 1 μL of plasma on average, ranging from 0.09 to 0.27). The cfDNA concentration of cancer patients (0.12 ng/μL on average) was obviously higher than that from normal controls (0.09 ng/μL on average: see details in Fig S1). After sequencing on the Illumina X Ten platform, raw data on of 4.21 G and 4.58 G on average were obtained from cancer patients and normal controls, respectively. The effective ratio of cancer patients (87.64% on average) was slightly higher than that in normal controls (84.18% on average). Average in normal controls (95.00%) was a bit higher than that in cancer patients (94.74%). Average GC contents were 42.90% and 43.94% in cancer patients and normal controls, respectively.

**Table 1:** Clinical characteristics and output data information

	Lung cancer patients	Normal controls
Sample size	41	40
Mean age (range) year	60 (44–75)	31 (21–66)
Mean concentration (range) ng/μL	0.12 (0.08–0.27)	0.09 (0.06–0.17)
Mean DNA volume (range) μL	64.51 (61.00–68.00)	64.60 (58.00–69.00)
Mean cfDNA amount (range) ng	7.91 (5.00–18.36)	5.81 (4.09–10.35)
Mean raw data (range) G	4.21 (2.08–5.44)	4.58 (3.16–8.44)
Mean effective ratio (range) %	87.64 (71.01–94.49)	84.18 (65.35–94.31)
Mean Q30 (range) %	94.74 (93.55–95.67)	95.00 (94.29–95.73)
Mean GC content (range) %	42.90 (41.72–43.75)	43.94 (42.45–46.56)

### Aneuploidy detection in plasma based on regular *z* score

**Figure 1:**
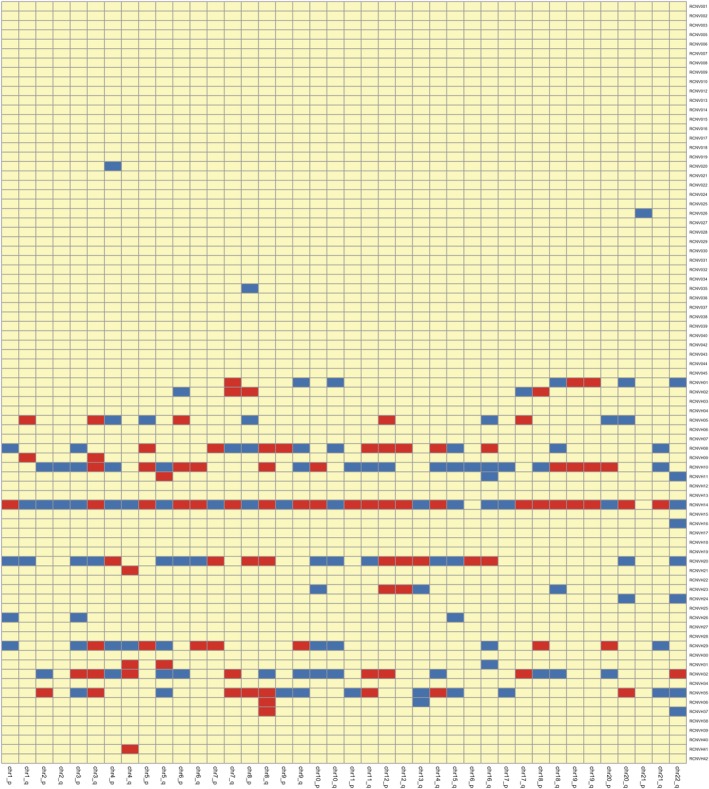
Heatmap of the arm‐level CNVs in each chromosome (x‐axis) of LC and normal controls (y‐axis). Deletions are marked in blue (*z* score < −2.96). Amplifications are marked in red (*z* score > 2.96). Alterations that are not significant are marked in yellow (−2.96 < z score < 2.96).

The *z* score has often been calculated to determine the difference in the percentage of mapped reads derived from plasma of cancer patients and that of normal controls.^11^, ^20^ Here, we chose chromosomal arm‐level CNVs because they usually occur approximately 30 times more frequently than focal CNVs (the focal CNVs are usually very short and occur at a frequency inversely related to their lengths).^10^ In this study, an absolute *z* score of ≥2.96 was determined to represent a statistically significant gain or loss of a chromosomal arm (Fig 1). Overall, 37 out of 40 normal controls were identified as not having any significant arm‐level alterations, yielding a specificity of 92.5%. However, nearly half of the cancer patients (20 out of 41) were identified as negative for the presence of arm‐level CNVs, yielding a sensitivity of as low as 51.2% (Table 2). In addition, among the 21 true‐positive results, 10 patients were in early stages (I and II), while 11 patients were in late stages (III and IV), indicating that prediction based on the regular *z* score algorithm in this study was not satisfactory for detecting early‐stage lung cancer detection.

**Table 2:** *Z*‐score based sensitivity and specificity to detect aneuploidy in plasma

	In total	Positive		Negative		Specificity %	Sensitivity %
		Early‐stage (I and II)	Late‐stage (III and IV)	Early‐stage (I and II)	Late‐stage (III and IV)	92.5 (37/40)	51.2 (21/41)
LC patients	41	10	11	16	4		
Normal controls	40	3	—	37	—		

### Analysis distribution of copy number alteration on chromosome arms

**Figure 2:**
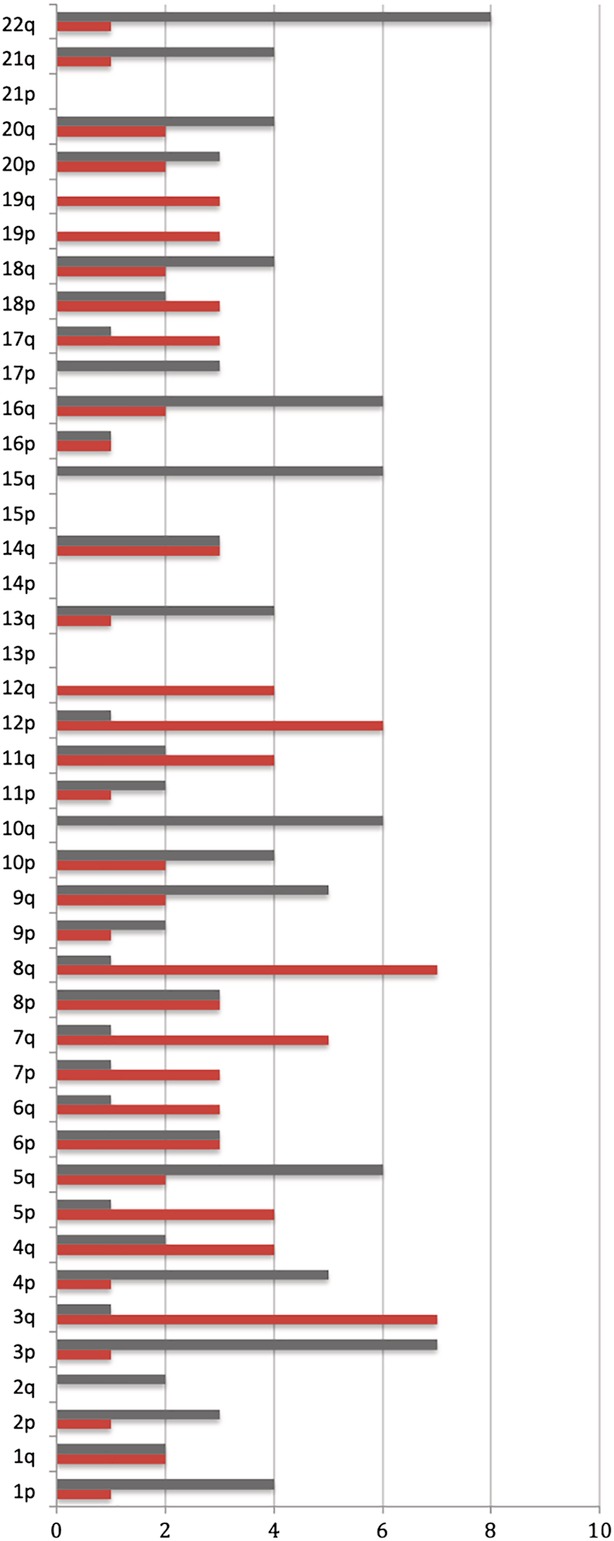
Column chart of the alteration frequency (x‐axis) of arm‐level CNVs in each chromosome (y‐axis) among all LC patients. Deletions are marked with gray columns, and amplifications are marked with red columns. For example, a total of four LC patients have deletions and one has amplifications on chromosome 1p.

Alterations of specific chromosomal regions are considered as a hallmark of different cancer types, which can be used for determining the diagnosis, prognosis, and impact of treatment.^21^, ^22^ We calculated the prevalence of CNVs on specific chromosome arms of 21 lung cancer patients in whom alterations had been detected from plasma. The number of patients with arm‐level alterations was counted for each chromosome arm (Fig 2), except for the short arm of the acrocentric chromosomes 22, X and Y. The results showed that amplifications tended to emerge on chromosomes 3q (7/21), 8q (7/21), 12p (6/21), and 7q (5/21). Deletions were frequently detected on chromosomes 22q (8/21), 3p (7/21), 5q (6/21), 16q (6/21), 10q (6/21), and 15q (6/21). As such, those chromosome arms mentioned above might be closely associated with the genesis and development of lung cancer.

### Individual sample prediction based on XGBoost

**Figure 3:**
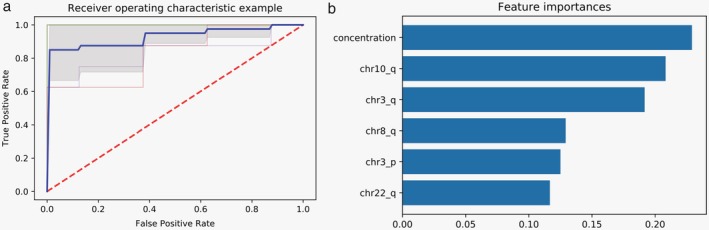
(**a**) ROC curve of five‐fold cross validation. (**b**) Six of the most important features selected by the model.

Although the prediction of LC patients based on regular *z* score in this study yielded a specificity of 92.5%, the sensitivity was far from satisfactory (51.2%). Considering the trend regarding the distribution of CNVs on each chromosome and cfDNA concentration variation during the process of cancer development, we applied the Extreme Gradient Boosting machine classifier, aiming to distinguish LC patients from normal controls using this noninvasive method. We randomly selected 56 samples (28 LC patients and 28 normal controls) to build up a training group for XGBoost machine learning classifier. The remaining 25 samples (13 LC patients and 12 normal controls) were selected as a test group. To validate this method, receiver operating characteristic (ROC) analysis was performed. For both training and test groups, the AUC was 1.00. ROC curve of five‐fold cross validation result are shown in Figure 3a. The top six features were selected according to the contribution in the model (Fig 3b). Concentration was ranked first, followed by chromosome arms 10q, 3q, 8q, 3p, and 22q. Based on these six features above, specificity and sensitivity of 100% were finally achieved in the test group.

### Result evaluation on model‐agnostic explanation method SHAP

**Figure 4:**
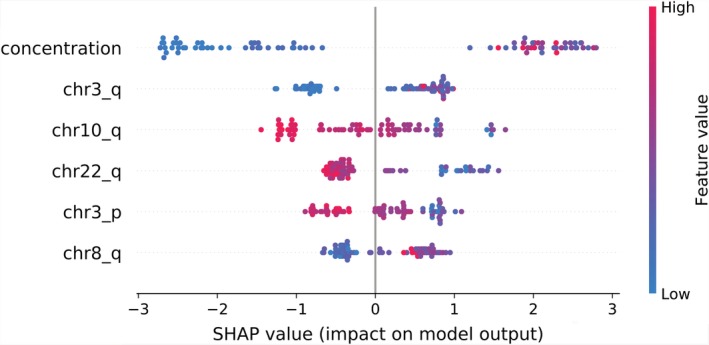
SHAP summary plots of top six features. The color represents the feature value (red, high; blue, low).

When considering the model accuracy, it is crucial to understand why a model makes a specific decision.^23^ SHAP is a unified framework for interpreting predictions by assigning each feature an importance value for a particular prediction.^19^ In a previous study, it was confirmed that SHAP values were consistent feature attributions by integrating them with an XGBoost model.^24^ Thus, in this study, we used the SHAP algorithm to obtain deeper insight into the top features depending on which decision the model made when predicting. The SHAP values in Figure 4 show the distribution of the impacts each feature had on the model output. The color represents the feature value (red high, blue low). This revealed that a higher concentration of cfDNA in plasma and CNV gains in chromosome arms 8q and 3q impacted on cancer pathogenesis. In contrast, CNV losses in chromosome arms 10q, 22q, and 3p were mainly associated with cancer pathogenesis. SHAP values for each feature are shown in Figure S2.

## Discussion

To our knowledge, this is the first study to choose the hot machine‐learning algorithm XGBoost as a classifier in lung cancer prediction. Chromosomal arm‐level CNVs in plasma were selected as biomarkers due to their frequent occurrence in the genome as well as noninvasive feasibility. In comparison with single nucleotide polymorphisms (SNPs), CNVs, the structural variations in the genome, have a greater effect and play a more important role in genetic variation, which is currently recognized as a risk factor in cancer etiology.^25^ With the rapid development and increasingly low cost of next generation sequencing (NGS) technology, sequencing‐based CNV detection has become increasingly favored in cancer studies. A previous study carried out a complete analysis of CNV detection under different conditions, indicating that coverage between 0.1× and 8× was associated with overall specificity between 91.7% and 99.9%, and sensitivity between 72.2% and 96.5%.^26^ Here, we set the whole genome sequencing to one‐fold as a robust depth, considering both the high occurrence frequency of arm‐level CNVs across the whole genome and the affordability cost of this low sequence depth for clinical application.

Initially, we used the regular *z* score to distinguish LC patients from normal controls and analyzed the CNVs profiles on each chromosome. However, the low sensitivity achieved with the *z* score was unsatisfactory. We then attempted to use the machine learning method XGBoost as a classifier. Through comparative analyses of *z* score and XGBoost, we consistently found that CNVs on chromosome 3p, 3q, 8q, 10q, and 22q which were among the top six features selected by XGBoost were also detected via the *z* score algorithm as the main affecting factors. Balsara and Testa^27^ found that among NSCLC samples the chromosome arms most frequently involved in gains included 3q, and those in losses included 3p and 22q, which was also supported by our study. Mermel *et al*.^28^ described a method that they developed to detect somatic copy‐number alterations in human cancers. When scoring whole chromosomal arm‐level events, they observed losses of chromosome 10. In addition, Petersen *et al*.^29^ performed comparative genomic hybridization (CGH) on 22 autoptic SCLCs to screen the tumor genome for genomic imbalances, and they observed deletions on chromosome 10q in 94% of tumors. Moreover, Kubokura *et al*.^30^ analyzed chromosome 8 copy numbers and c‐myc gene amplification in non‐small cell lung cancer, and they found that the number of chromosome 8 aberrations was significantly lower in patients who survived for five years or more. Their study suggested that the rate of chromosome 8 aberration is an additional prognostic factor of NSCLC patients. This previous research supports the assertion that the classifier applied in our study is reasonable and interpretable. Classification and data mining methods have become a particular focus of interest in the medical field due to their value in diagnostic and analytical decision‐making. Many algorithms for cancer prediction, such as Support Vector Machines (SVMs),^31^ Artificial Neural Networks (ANNs),^32^ and Bayesian Networks (BNs),^33^, ^34^ were applied in previous studies. With the application of machine learning methods, the accuracy of cancer prediction outcome has significantly improved by 15%–20%.^35^ However, building accurate and computationally efficient classifiers for medical use is a major challenge. Asri *et al*.^36^ compared four different algorithms for breast cancer prediction, and the results showed that SVM performed the best in terms of achieving the highest accuracy (97.13%) with the lowest error rate. Tian *et al*.^37^ focused on esophageal cancer diagnosis and utilized 5hmC characteristics detected in cfDNA as a biomarker. For cancer classification, they used the XGBoost method in 333 samples, including 177 healthy controls plus six replicative samples and 150 esophagus cancer patients, and achieved a sensitivity of 93.75% and specificity of 85.71% (AUC = 0.972). XGBoost has gained popularity by winning numerous machine‐learning competitions. Nielsen^38^ attempted to explain XGBoost's many advantages over other methods. He indicated that first tree boosting can take the bias‐variance tradeoff into consideration during model fitting, and XGBoost deals with the bias‐variance tradeoff even more meticulously by introducing some subtle improvements. At the beginning of our study, the *z* score based classification of cancer patients and normal controls failed due to its low sensitivity. Thus we chose the efficient machine learning method, XGBoost as a classifier, hoping to achieve significant promotion. Finally, beyond our expectations, we achieved specificity and sensitivity of 100% in our limited sample set. As mentioned above, the small sample size as noted in many other similar studies is an obvious limitation here. The larger the data set, the more likely it is to lead to reasonable validation of the estimators.^39^ Thus, in future work, we plan to perform studies on larger cohorts from multiple‐centers.

## Disclosure

The authors declare that there are no conflicts of interests.

## Supporting information


**Figure S1** CfDNA concentration of cancer patients and normal people.

**Figure S2** SHAP values for each feature.

**Table S1** Detailed information of the cohort.
