# Supplementary figures and images for: Copy number variation in plasma as a tool for lung cancer prediction using Extreme Gradient Boosting (XGBoost) classifier
Source: Thorac Cancer. 2019 Nov 6;11(1):95–102. doi: 10.1111/1759-7714.13204 (PMC6938748; doi:10.1111/1759-7714.13204)

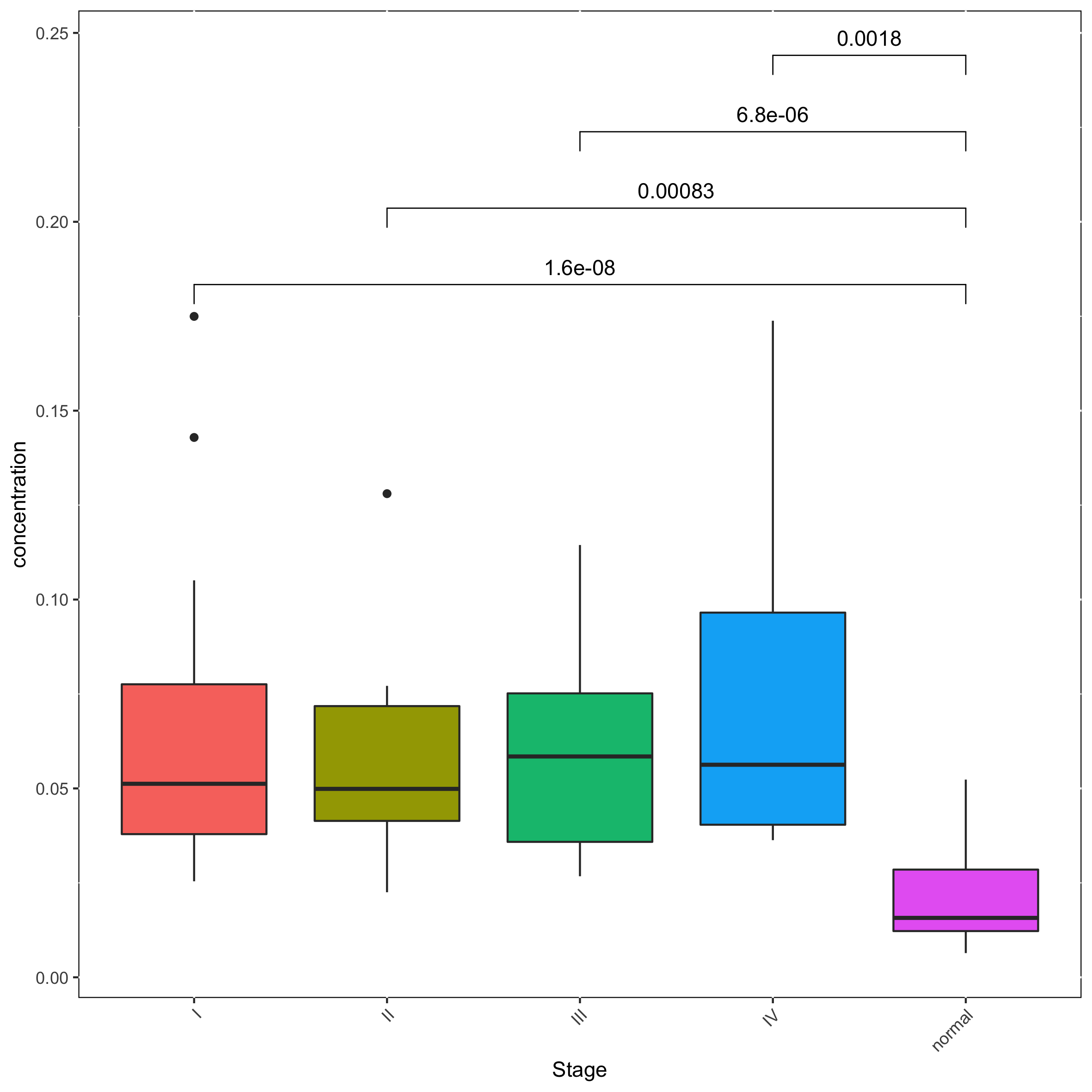

Supplement: Supplementary file 1 — Figure S1 CfDNA concentration of cancer patients and normal people. [file TCA-11-95-s001.png]

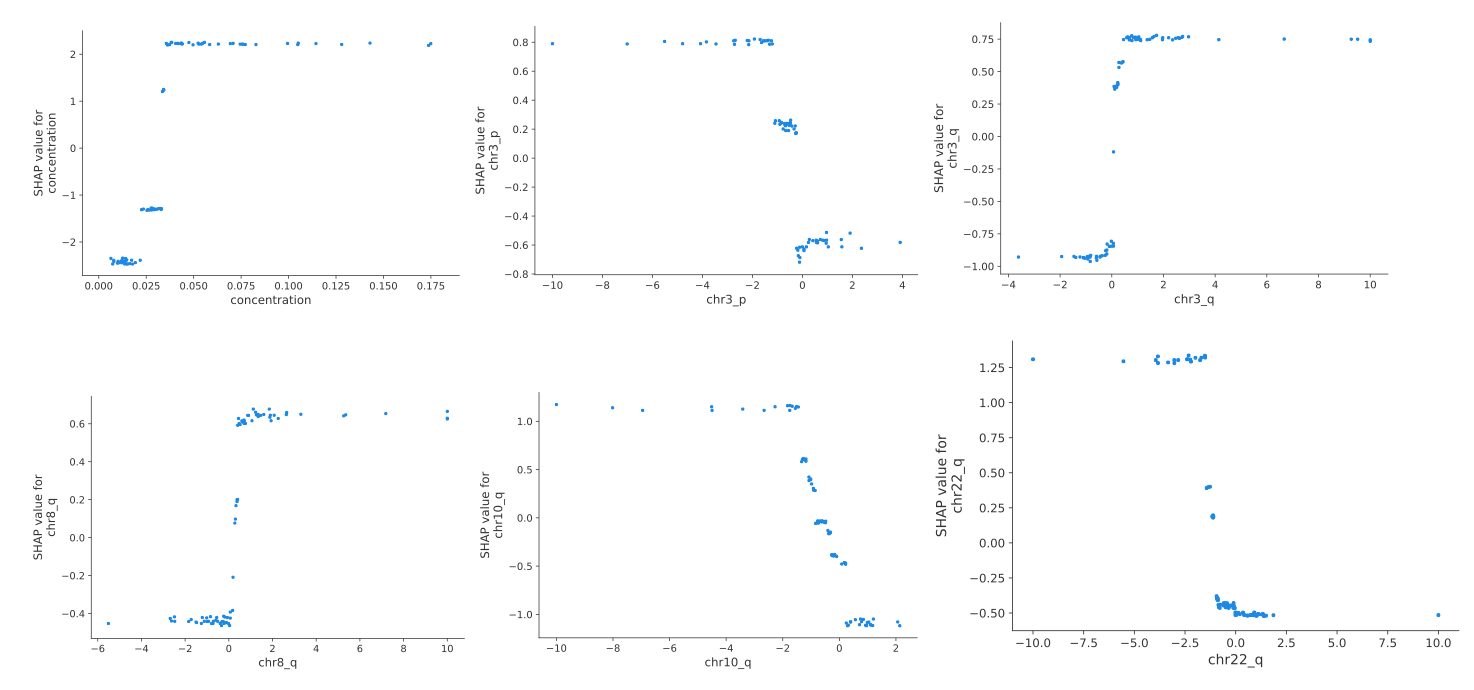

Supplement: Supplementary file 2 — Figure S2 SHAP values for each feature. [file TCA-11-95-s003.png]
